# Effect of ZnO nanoparticles on methicillin, vancomycin, linezolid resistance and biofilm formation in *Staphylococcus aureus* isolates

**DOI:** 10.1186/s12941-021-00459-2

**Published:** 2021-08-21

**Authors:** Wedad M. Abdelraheem, Rasha M. M. Khairy, Alaa I. Zaki, Shaimaa H. Zaki

**Affiliations:** Department of Microbiology and Immunology, Faculty of Medicine, Minia University, Minia, 61511 Egypt

**Keywords:** MRSA, VRSA, LRSA, Biofilm, ZnO-NPs

## Abstract

**Background:**

Multidrug resistant (MDR) and biofilm producing *Staphylococcus aureus* strains are usually associated with serious infections. This study aimed to evaluate the antibacterial and antibiofilm-formation effects of zinc oxide nanoparticles (ZnO-NPs) against *staphylococcus aureus* (*S. aureus)* isolates.

**Methods:**

A total of 116 *S. aureus* isolates were recovered from 250 burn wound samples. The antimicrobial/antibiofilm effects of ZnO-NPs against methicillin, vancomycin and linezolid resistant *S. aureus* (MRSA, VRSA and LRSA) isolates were examined using phenotypic and genotypic methods. The minimum inhibitory concentration (MIC) of ZnO-NPs was determined by microdilution method. The effects of sub-MIC concentrations of ZnO-NPs on biofilm formation and drug resistance in *S. aureus* were determined by the microtiter plate method. The change in the expression levels of the biofilm encoding genes and resistance genes in *S. aureus* isolates after treatment with ZnO-NPs was assessed by real time reverse transcriptase PCR (rt-PCR).

**Results:**

MICs of ZnO-NPs in *S. aureus* isolates were (128–2048 µg/ml). The sub-MIC of ZnO-NPs significantly reduced biofilm formation rate (the highest inhibition rate was 76.47% at 1024  µg/ml) and the expression levels of biofilm genes (*ica A, ica D* and *fnb A)* with P < 0.001. Moreover, Sub-MIC of ZnO-NPs significantly reduced the rates of MRSA from 81.9 (95 isolates) to 13.30% (15 isolates), VRSA from 33.60 (39 isolates) to 0% and LARSA from 29.30 (34) to 0% as well as the expression levels of resistance genes (*mec A, van A* and *cfr*) with P value < 0.001.

**Conclusion:**

ZnO-NPs can be used as antibiofilm and potent antimicrobial against MRSA, VRSA and LRSA isolates.

**Supplementary Information:**

The online version contains supplementary material available at 10.1186/s12941-021-00459-2.

## Background

*Staphylococcus aureus* (*S. aureus*) is an important human pathogen which cause a variety of clinical infections [1]. In the past few decades, the treatment of infections caused by *S. aureus* had become a big challenge due to emergence of multi-drug resistant strains such as Methicillin-Resistant *Staphylococcus aureus* (MRSA) in community and hospital settings [2]. Methicillin resistance is caused by *mecA* or *mecC* gene, encoding penicillin-binding protein (PBP2a) or (PBP2ALGA) with low affinity for β-lactams [3]. For treatment of MRSA, vancomycin was used as the drug of choice for decades [4]. However, vancomycin resistant *S. aureus* (VRSA) isolates and vancomycin intermediate resistant *S. aureus* (VISA) have emerged [5]. Vancomycin resistant *S. aureus* (VRSA) is mediated by *van*A gene cluster, which is transmitted by vancomycin resistant enterococci [4, 6]. Linezolid is an oxazolidinone which has become a good alternative to vancomycin in the treatment of infections caused by gram-positive organisms, including VRSA or VISA isolates [7]. Unfortunately, Linezolid resistance among *S. aureus* isolates (LRSA) was reported in USA shortly after its use [8]. Development of Linezolid resistance in these early studies was due to mutation in the 23S rRNA gene, but it seemed to be an uncommon finding [9, 10]. However, a different mechanism of Linezolid resistance has reported: the acquisition of plasmid-mediated ribosomal methyltransferase *cfr* gene, which also mediates chloramphenicol resistance [11, 12]. In addition to development of multi-drug resistance among *S. aureus* strains, biofilm formation is reported as an important cause of treatment failure and recurrent infections [13]. Biofilm formation by *S. aureus* is encoded by (*ica*) ADBC genes which mediate synthesis of polysaccharide intracellular adhesin (PIA) [14]. Therefore, detection of one or more of these genes can determine the ability of *S. aureus* strains to produce biofilm [15]. Biofilm protects the organism from antimicrobials and also from killing by the host immune system [13]. With the emergence, spread, and persistence of resistance to different antimicrobials, the development of novel and effective alternatives to the traditional antibiotics has become an urgent need. The progressions in nanotechnology hold a promising future of nanomaterials as antimicrobial agents. Nanomaterials have a broader microbicidal spectrum than traditional antibiotics [16]. ZnO nanoparticles (ZnO-NPs) have been identified as one of the most promising metallic nanomaterials. In recent years, there is an increasing interest in ZnO-NPs as effective antibacterial agents due to their safety and stability for human cells [17, 18]. The current study aimed to use phenotypic and molecular methods to assess the efficacy of ZnO-NPs against MRSA, VRSA, LRSA and biofilm formation among *S. aureus* isolated from burn wounds.

## Materials and methods

In this cross sectional study, a total of 250 burn wound samples of 250 burn patients who attending outpatient’s clinics of the plastic surgery department, Minia university hospital were collected from April 2019 to December 2019. Patients with clinical findings of burn wound infection, such as erythema, swelling and sepsis were included. This study was carried following the guidelines of the declaration of Helsinki and approved by the Medical Ethics Committee of Faculty of medicine, Minia University, Egypt. Informed consent was obtained from each participant.

### Bacterial isolation

Out of 250 samples, 116 *S. aureus* isolates were identified according to the standard methods using Gram staining, catalase test, tube coagulase test, DNase agar and cultivation on mannitol salt agar. *Staphylococcus aureus* isolates were confirmed by identification of *16 s RNA* gene expression among all 116 *S. aureus* isolates. Confirmed *S. aureus* isolates were kept in trypticase soy broth with sterilized 15% glycerol at − 20 °C.

### Antibacterial activity of ZnO-NPs

ZnO-NPs with an average particle size of 30 nm and purity of above 99% was used in the study (Sigma Aldrich, St. Louis, MO, USA). Stock solution of ZnO-NPs was prepared by dissolving ZnO-NPs in propylene glycol in the highest concentration (1000 μg/ml). MIC values of ZnO-NPs for all *S. aureus* isolates were determined by broth micro-dilution method using sterile 96-well microplates. Gradient concentrations of ZnO-NPs (0.50–4096 μg/ml) were inoculated with 100 μl of bacterial suspension of each isolate with turbidity equivalent to 0.5 Mc-Farland in the tubes. Tubes with culture media and microbial suspension without nanoparticles were used as positive control and tubes with sterile broth were used as negative control. The plates were incubated overnight at 37 °C. MIC is the lowest concentration of the ZnO-NPs that inhibit visible bacterial growth [19]. After identification of MIC value for each isolate, sub-inhibitory concentrations (1/2 MIC) were calculated. All experiments were carried out three times.

### Phenotypic identification of MRSA, VRSA and LRSA using micro-dilution method

MICs of oxacillin, vancomycin and linezolid in *S. aureus* isolates were determined by micro-dilution method using sterile 96-well microplates. Commercial oxacillin, vancomycin hydrochloride (MYLAN S.A.S Company, France) and linezolid infusion (Averroes pharma company, Egypt) were prepared in the highest concentration to be used. The MICs of each antimicrobial agent were determined and interpreted according to CLSI 2019 guidelines [20].

### Molecular identification of MRSA, VRSA and LRSA

Bacterial RNA was extracted by Easy-spin™ Total RNA Extraction Kit (iNtRON biotechnology, South Korea) and lysozyme 10 mg/ml from *S. aureus* isolates according to the manufacturer’s instructions. Gene expression of antimicrobial resistance genes (*mec A* for methicillin, *van A* for vancomycin and *cfr* for Linezolid) were tested using quantitative real-time reverse transcriptase-polymerase chain reaction (rt-PCR).* 16 s RNA* gene was used as a reference gene. One step Sybr green kits (SensiFAST SYBR Lo-ROX Kit, Meridian Life science, UK) were used according to manufacturer’s instructions. Primers used in the study were listed in Table 1. Each rt-PCR reaction was prepared with a final volume of 20 µg (master mix: 10 µg, Forward primer: 0.8 µg, Reverse primer: 0.8 µg, Reverse transcriptase: 0.2 µg, RNase inhibitor, 0.4 µg, Water up to 16 µg and template: 4 µg). Negative control samples contain deionized water instead of template were used with each run. The conditions of the different reactions were adjusted according to kits protocol as follows: reverse transcription for 10 min at 45 °C, Polymerase activation for 2 min at 95 °C, then 40 cycles of denaturation for 5 s at 95 °C and annealing/extension for 20 s at 60 °C. PCR products were analyzed by gel electrophoresis, to exclude any unspecific products.

**Table 1:** The Primers sequence of the tested genes

Gene	Sequence	References
*icaA*	F:5′-ACACTTGCTGGCGCAGTCAA-3′	[21]
	R:5′-TCTGGAACCAACATCCAACA-3′	
*icaB*	F:5′-AGAATCGTGAAGTATAGAAAATT-3′	[22]
	R:5′-TCTAATCTTTTTCATGGAATCCGT-3′	
*icaD*	F:5′-ATGGTCAAGCCCAGACAGAG-3′	[23]
	R:5′- AGTATTTTCAATGTTTAAAGCAA-3′	
*fnbA*	F:5′-CATAAATTGGGAGCAGCATCA-3′	[24]
	R:5′-ATCAGCAGCTGAATTCCCATT-3′	
*mecA*	F:5′-GTAGAAATGACTGAACGTCCGATAA-3′	[25]
	R:5′-CCAATTCCACATTGTTTCGGTCTAA-3′	
*vanA*	F:5′-CATGAATAGAATAAAAGTTGCAATA-3′	[26]
	R:5′-CCCCTTTAACGCTAATACGACGATCAA-3′	
*cfr*	F:5-TGAAGTATAAAGCAGGTTGGGAGTCA3′	[26]
	R: 5′-ACCATATAATTGACCACAAGCAGC-3′	
*16S rRNA*	F:5′-GTA GGT GGC AAG CGT TAT CC-3′	[27]
	R:5′-CGCACATCAGCGTCAG-3′	

### Biofilm formation testing among *S. aureus* isolates

The isolated organisms were tested for their ability to form biofilm as previously described [28]. Each isolate was inoculated into trypticase-soy broth and incubated overnight. After adjusting the turbidity of bacterial suspensions to the turbidity of 0.5 McFarland, 100 μl of each isolate was inoculated into sterile 96 well microtiter plate except last column that used as negative control. The inoculated plate was incubated for 24 h. The contents of wells were gently decanted and washed by saline. The wells were stained by 150 μl of crystal violet (0.2%) for 15 min at room temperature. The stain was gently discarded and wells were washed by water. The plate was dried at room temperature and the crystal violet in stained cells was solubilized with 95% ethanol. The optical density (OD) of each well was measured at 620 nm by ELISA reader. The average OD values were calculated for all tested isolates and negative controls. The isolates were divided into four categories non biofilm, weak, moderate and strong biofilm producer as previously described [28].

### Molecular identification of biofilm formation among *S. aureus* isolates

Gene expression of biofilm encoding genes (*ica A, ica B, ica D* and *fnb A*) were tested using quantitative real-time reverse transcriptase-polymerase chain reaction (rt-PCR).* 16 s RNA* gene was used as a reference gene. One step Sybr green kits (SensiFAST SYBR Lo-ROX Kit, Meridian Life science, UK) were used according to manufacturer’s instructions. Primers used were listed in Table 1

### Phenotypic identification of MRSA, VRSA and LRSA after application of ZnO-NPs

Sub-inhibitory concentration of ZnO-NPs (1/2 MIC) of each sample was measured and added to wells that inoculated by bacterial broth adjusted to the turbidity of 0.5 McFarland standard then, the plates were incubated at 37 °C for 24 h. Micro-dilution tests for oxacillin, vancomycin and linezolid were repeated with the same previous steps to all ZnO-NPs treated isolates and MICs were determined according to CLSI 2019 guidelines [20].

### Biofilm formation testing among *S. aureus* isolates after ZnO-NPs application

Biofilm-forming isolates were inoculated in trypticase-soy broth and incubated 24 h at 37 °C. About 100 μl of each isolate’ suspension was inoculated into sterile 96 well microtiter plate and mixed with 100 μl of 1/2 MIC of ZnO-NPs. The microplate was incubated at 37 °C until the biofilm formation. The results were interpreted by ELISA plate reader at 620 nm using the same steps that used before.

### Effect of ZnO-NPs on genes expression

Gene expression of biofilm encoding genes (*ica A, ica B, ica D* and *fnb A*) and antimicrobial resistance genes (*mec A* for methicillin, *van A* for vancomycin and *cfr* for Linezolid) were tested using quantitative real-time reverse transcriptase-polymerase chain reaction (rt-PCR) after treating of *S. aureus* isolates with sub-inhibitory concentration of ZnO-NPs (1/2 MIC) of each sample and incubating the plates at 37 °C for 24 h, bacterial RNA was extracted again using the same method that used before. Gene expression of biofilm encoding genes and antimicrobial resistance genes were tested again using the same method that used before. PCR products were analyzed by gel electrophoresis, to exclude any unspecific products. The relative expression of target genes was calculated using the equation; RQ = 2^−ΔΔCt^ as described previously [29].

### Statistical analysis

All data collected in this study were stored in a computer database. Statistical analysis was done on SPSS package version 23.0 (SPSS Inc., Chicago, IL, USA). Chi-squared tests were performed for categorical data, while Mann Whitney *U* test and *Z* test were performed for comparison of continuous data. Roc curve analysis was used to detect specificity and sensitivity of the used methods.

## Results

### Isolation of *S. aureus*

Out of 250 wound samples, 116 (46.40%) isolates were identified as *S. aureus.*

### Antibacterial activity of ZnO-NPs

**Fig.1:**
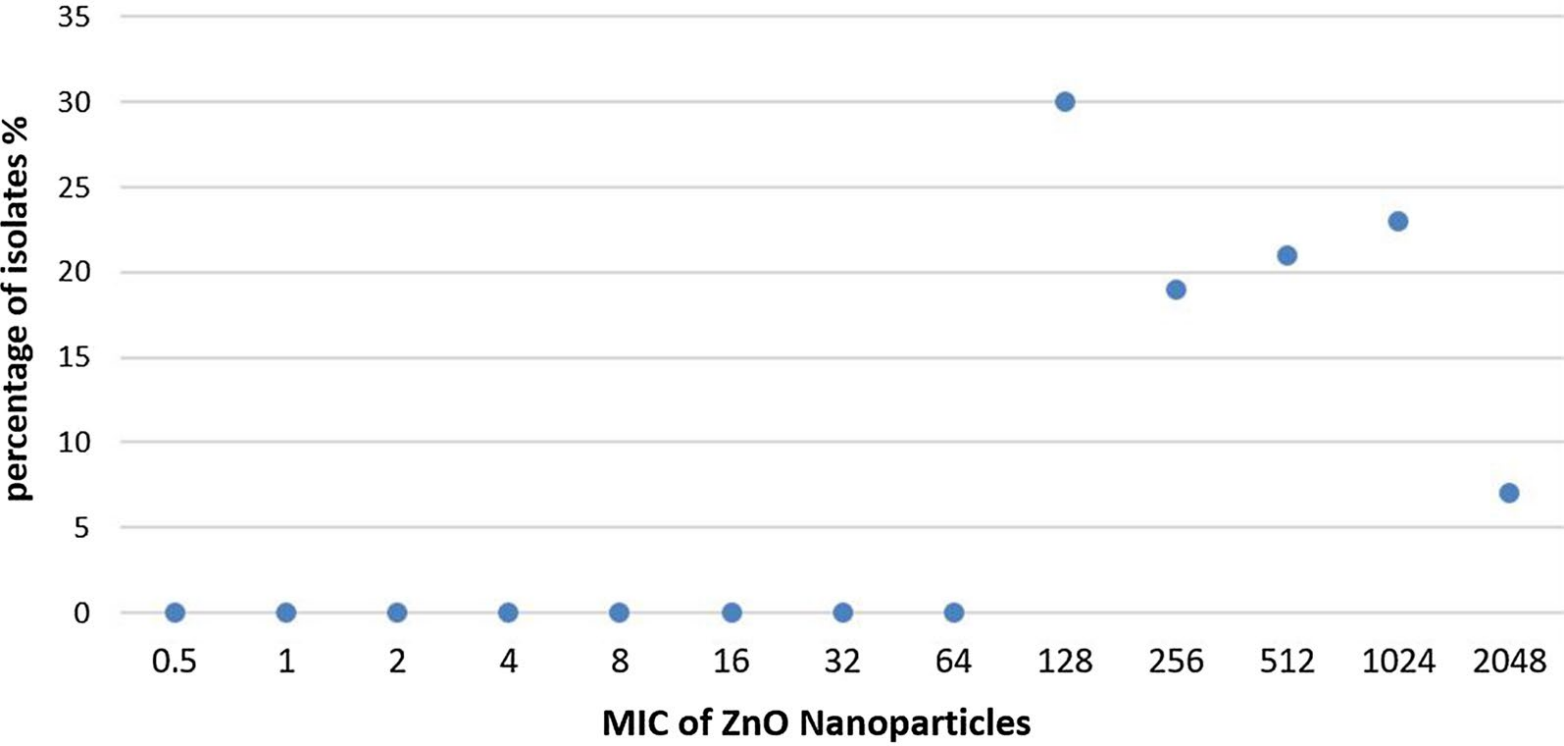
MICs of ZnO-NPs in all *S. aureus* isolates

MICs of ZnO-NPs among *S. aureus* isolates were (128–2048 µg/ml) as presented in Fig. 1.

### Effect of ZnO-NPs on biofilm formation

**Fig. 2:**
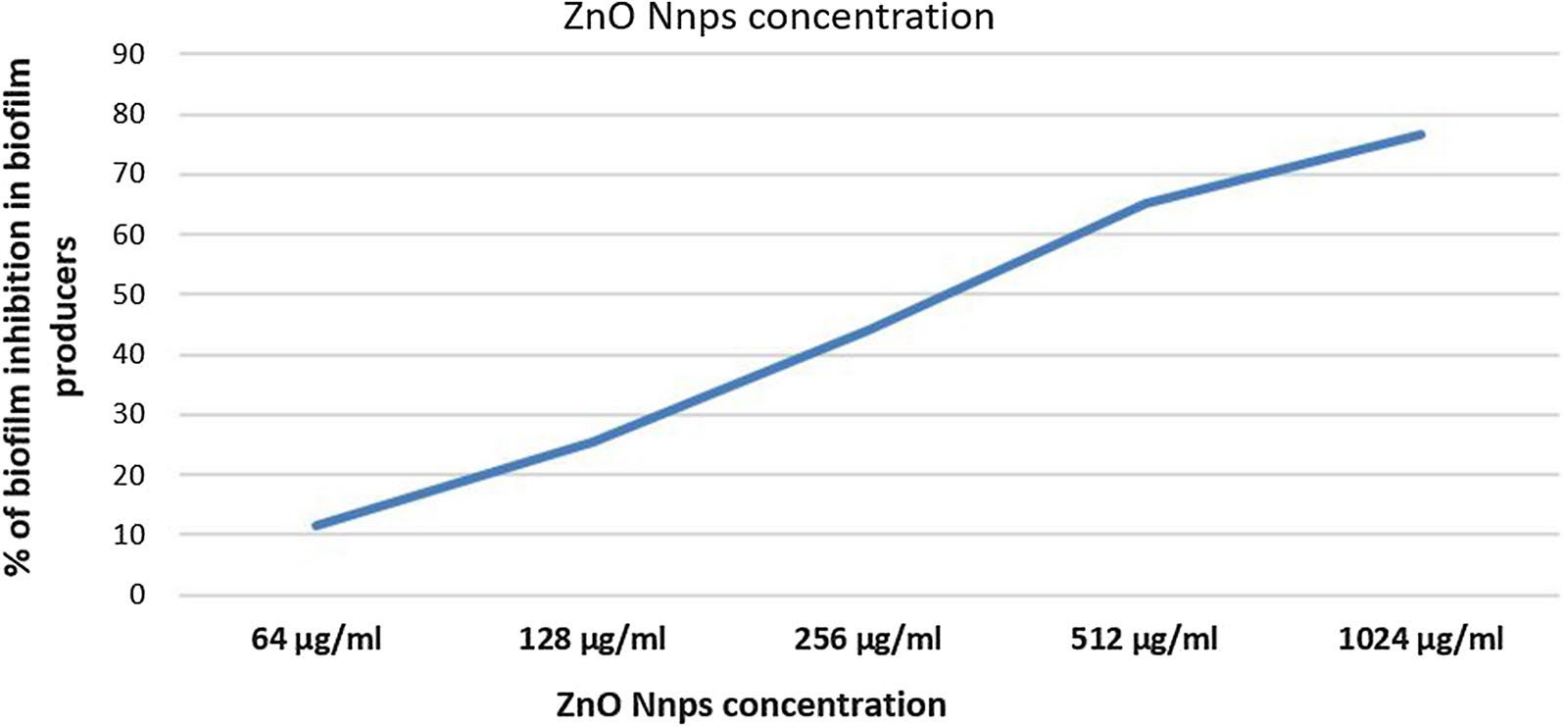
Correlation between biofilm inhibition and ZnO-NPs concentrations

All isolates of *S. aureus* (116) were tested for their ability to form biofilm using microtiter plate; 31/116 (26.70%) were non-biofilm producers and 85 (73.30%) were biofilm producers (weak: 44/116 (38%); moderate 32/116 (28%); and strong: 9/116 (8%). The frequencies MRSA, VRSA and LRSA among biofilm and non-biofilm producers were presented in Table 2. All biofilm producers (85) were tested for their ability to form biofilm after treating with 1/2 MIC of ZnO-NPs of each isolate. ZnO-NPs at a concentration of 1024ug/ml could inhibit biofilm formation in 76.47% (65/85) of biofilm producing isolates. There was a positive correlation between concentrations of ZnO-NPs and the rates of biofilm formation inhibition (Fig. 2).

**Table 2:** The frequencies MRSA, VRSA and LRSA among biofilm and non-biofilm producers

Antibiotic resistance	Biofilm production				Chi-squared	
	Non-biofilm producer N = 30		Biofilm producer N = 86		X^2^	p-value
	Resistance freq. (%)	Sensitivity freq. (%)	Resistance freq. (%)	Sensitivity freq. (%)		
Oxacillin	23 (76.6%)	7 (23.4%)	72 (83.72%)	14 (16.28%)	0.747	0.388
Vancomycin	17 (56.6%)	13 (43.4%)	22 (25.58%)	64 (74.42%)	10.549	0.005
Linezolid	14 (46.66%)	16 (53.34%)	20 (23.25%)	66 (76.75%)	5.883	0.015

P-value ≤ 0.05 is significant

### Effect of ZnO-NPs on MRSA, VRSA and LRSA among *S. aureus* isolates

Micro-dilution method was used to identify MRSA, VRSA and LRSA. The prevalence of MRSA (isolates with MIC of ≥ 4 μg/mL for oxacillin) was 95/116 (82%). The prevalence of VRSA (isolates with MIC ≥ 16 μg/mL to vancomycin) was 39/116 (34%) and VISA (isolates with MIC 4–8 ug/mL to vancomycin) was 6/116 (5.17%). The prevalence of LARSA (isolates with M IC ≥ 8 μg/mL to linezolid) was 34/116 (29.3%).

**Fig. 3:**
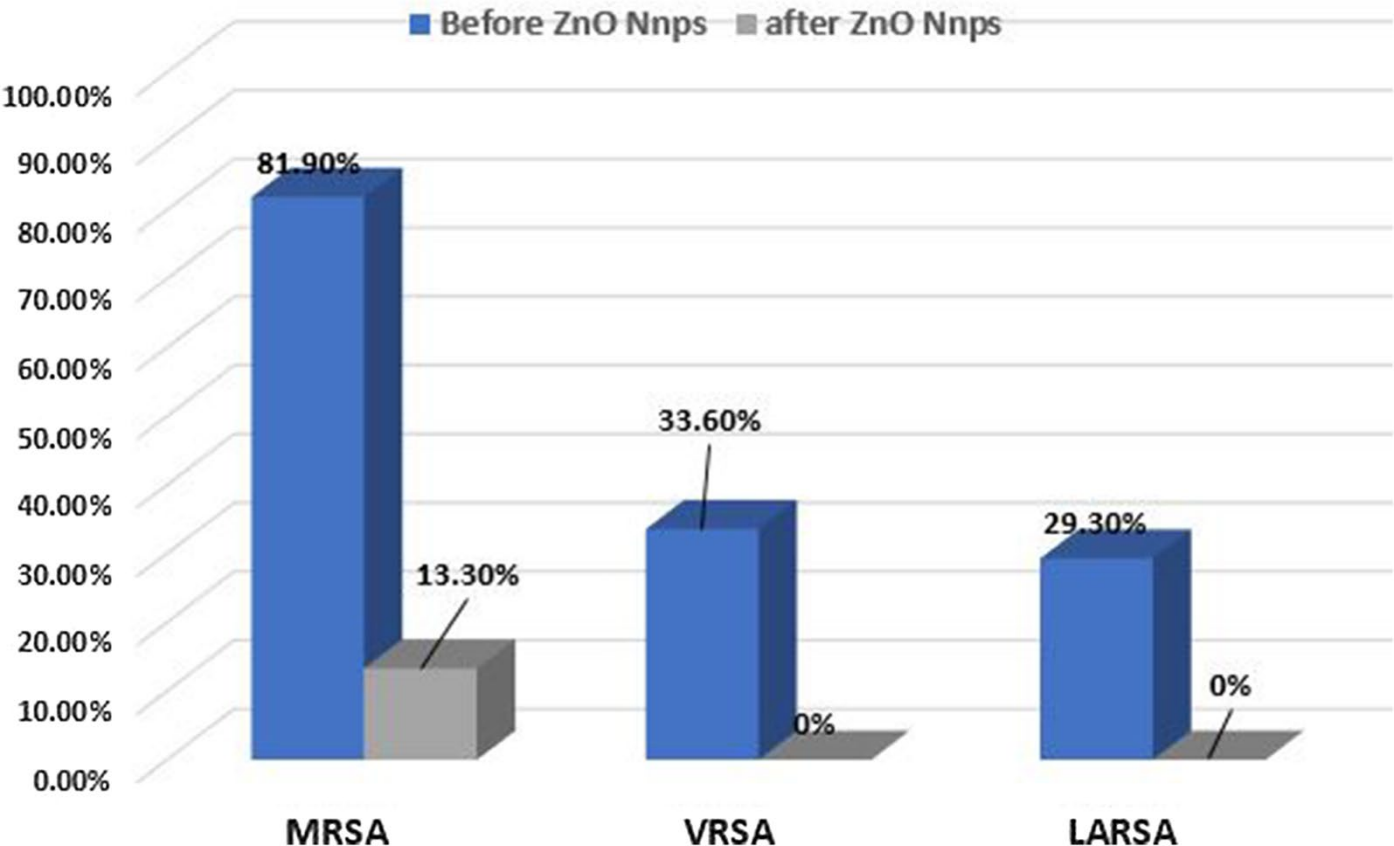
MRSA, VRSA and LRSA prevalence before and after ZnO-NPs application

MICs identification was repeated after treatment of all isolates with 1/2 MIC of ZnO-NPs. MRSA decreased from 81.9 (95 isolates) to 13.3% (15 isolates), VRSA decreased from 33.60 (39 isolates) to 0% and LARSA decreased from 29.30 (34) to 0% (Fig. 3). This decrease was statistically significant as p values were ≤ 0.005.

### Effect of ZnO-NPs on genes expression

**Fig. 4:**
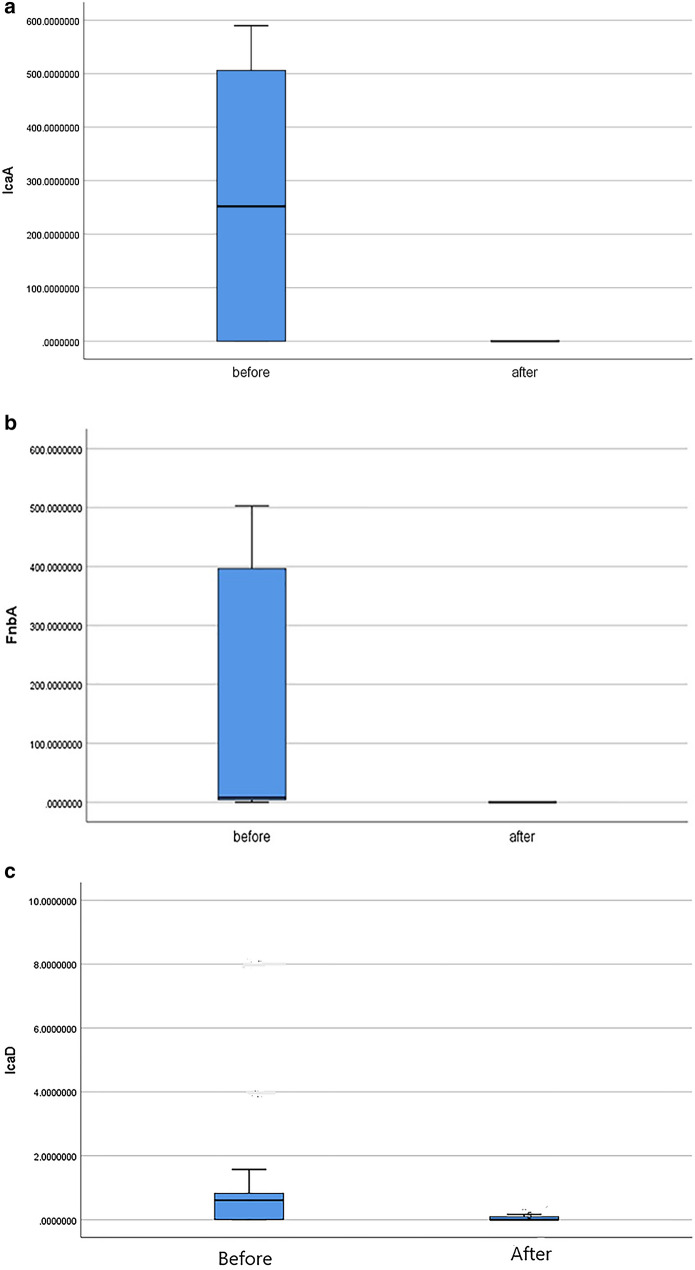
**a** Expression of *icaA* gene before and after ZnO-NPs application, P-value = 0.005. **b** Expression of *icaD* gene before and after ZnO-NPs application, P-value = 0.005. **c** Expression of *fnb A* gene before and after ZnO-NPs application, P-value = 0.008

**Fig. 5:**
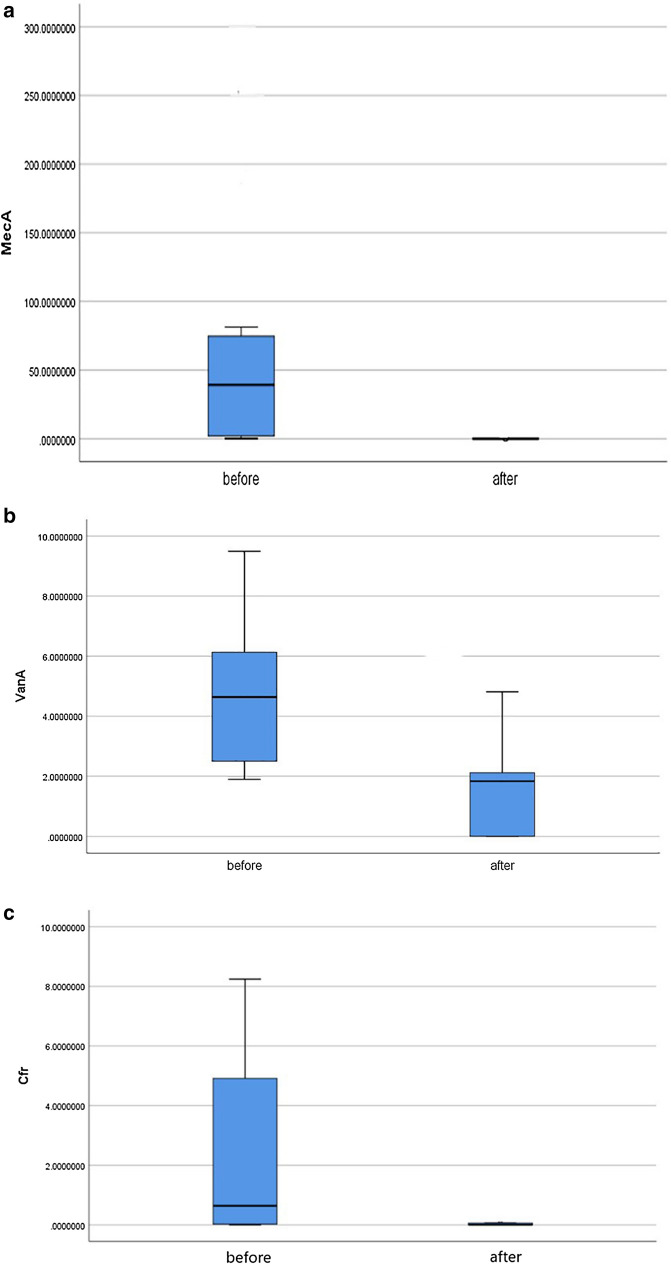
**a** Expression of *MecA* gene before and after ZnO-NPs application, P-value = 0.005. **b** Expression of *vanA* gene before and after ZnO-NPs application, P-value = 0.001. **c** Expression of *cfr* gene before and after ZnO-NPs application, P-value = 0.002

Using real time RT-PCR; *ica A* gene and *fnb A* gene expressed in 116 isolates (100%) of *S. aureus* isolates and *ica D* expressed in 96 isolates (82.7%). However *ica B* gene was not detected in the study isolates. Regarding antimicrobial resistance genes, the rate of *mec A* expression among *S. aureus* isolates was (94 isolates; 81%), *van A* was (22 isolates; 18.96%) and *cfr* gene was (29 isolates; 25%). Antimicrobial resistance genes were not detected in sensitive or intermediate resistant isolates. Analyzing expression levels of biofilm encoding genes (*ica A, ica B, ica D* and *fnb A*) among *S****.**** aureus* isolates compared to their expression levels after treating of the isolates with ZnO-NPs using Mann–Whitney *U*-test revealed that; there was statistically significant decrease of the expression levels of *ica A, ica D* and *fnb A* genes after treating of the *S. aureus* isolates with ZnO-NPs (P < 0.001, for each) (Fig. 4a–c; Additional file 1: Table S1). As regards to antimicrobial resistance genes (*mec A, van A* and *cfr*); the expression levels of the three genes significantly decreased after treating of *S. aureus* isolates with ZnO-NPs (P < 0.001, for each) as shown in (Fig. 5a–c). The receiver operating characteristic curve (ROC) analysis using the expression levels of the studied genes was used to assess the accuracy of these results and revealed that; there were highly significant decreases in the expression levels of the studied genes after treating isolates with sub-MICs of ZnO-NPs with high sensitivity and specificity as shown in (Table 3).

\

**Table 3:** ROC curve analysis

Gene	Area under the curve	95% confidence interval	P value	Sensitivity (%)	Specificity (%)
*fnb A*	0.83	0.65–1.00	0.01	71.70	85.00
*ica A*	0.79	0.62–0.95	0.01	66.70	83.00
*ica D*	0.84	0.73–0.95	0.01	66.00	83.30
*mec A*	0.93	0.85–1.00	0.01	88.00	97.00
*VanA*	0.893	0.758–1	0.002	76	92
*Cfr*	0.859	0.721–0.997	0.001	62	87

## Discussion

The multidrug resistant strains of *S. aureus* are increasing, making the treatment more difficult. The prevalence of MRSA among *S. aureus* isolates is the highest in Egypt compared to other African countries [30]. The prevalence of MRSA in Egypt is ranging from 24 to 82% [31], which is comparable with the prevalence reported in the current study (95/116, 81.90%). Our prevalence is comparable also with other reports from developing countries; (80%) [32], 75% [33] and 76% [34]. The most effective drugs against MRSA are vancomycin and linezolid [35], however, isolates with reduced susceptibility to vancomycin are increasing [36]. The global prevalence of VRSA and VISA before 2010 was 1.2% and after 2010 were 2.40% and 4.3% respectively and the prevalence of VRSA in Egypt was 5.50% [37]. However, the current study has reported a high prevalence of VRSA and VISA; 33.62% (39/116) and 5% (6/116) respectively, that agrees with a similar report from Egypt [33] where the author reported that 20.68% of the isolates were VISA, and 20.68% were VRSA. In Egypt, yet a few researches studied the resistance against linezolid among staphylococcal isolates using phenotypic methods only, where the prevalence of LRSA ranged from 5 to 15.4% [38, 39]. The prevalence of LRSA in the current study was (34/116, 29.31%), that is higher than the previous Egyptian reports. Harcharan singh et al. also reported a high percentage of LRSA in Rajasthan (20.3%) [40]. On the other context, the current finding is higher than the global rates reported in the LEADER or ZAAPS studies [41, 42]. This could be due to the availability of linezolid in the Egyptian market, its use as an empiric treatment in our locality and absence of guidelines that control its use. One of the main reasons of antibiotic resistance is the rebellious nature of biofilms produced by these pathogens. In this study, 85/116 (73%) of isolates were identified as biofilm producers, that is compatible with other studies investigated biofilm production by *S. aureus* isolated from wound samples [43, 44]. Our study focused on the development of promising alternative agents for treatment of these serious infections such as ZnO-NPs. Interestingly, ZnO-NPs were identified by several reports as non-toxic to human cells [45]. ZnO-NPs should penetrate into bacterial cells to express the antibacterial activity [46]. Therefore, the broth dilution assay can be considered as accurate and confirmative method for identification of antibacterial activity of ZnO-NPs [47]. By using the broth dilution method, our study showed that, MICs of ZnO-NPs among *S. aureus* isolates were ranging from 128 to 2048 µg/ml. Other studies have also reported that bactericidal effect of ZnO-NPs is concentration-dependent [48–50]. By use of sub MICs of ZnO-NPs (68–1000 µg/ml), the biofilm formation among the study isolates was decreased up to 76.47% (65/85). This was comparatively higher than that used by Jesline et al. who detected (100/200/500 µg/ml) concentrations were able to inhibit bacterial growth and biofilm formation of all *S. aureus* isolates [51] and lower than that used by Jasim et al. who observed that, the highest rate of biofilm inhibition among VRSA was 73.95 ± 2.17% at 10.00 µg/ml of ZnO-NPs [52]. Similar results were reported by Mahamuni et al., who reported 67.3% biofilm inhibition [53] and Abd El-Hamid who reported a percentage of (99.73%) of biofilm inhibition among S. aureus isolates [54]. With the use of sub MICs of ZnO-NPs on *S. aureus* isolates, resistance to oxacillin (MRSA) decreased from 81.90 to 13.30%, VRSA decreased from 33.60 to 0% and, LRSA decreased from 29.30 to 0%. Using *Z* test, the decrease in MRSA, VRSA and LRSA was statistically significant as Z score were 11.10, 6.85 and 6.47 respectively (all these values are significant). These findings agree with Ghazi and Alsammak, who observed that the efficacy of vancomycin was improved in combination with ZnO nanoparticles (MICs of vancomycin decreased from (2500–5000 μg/mL) to (39–78.13 μg/mL) when mixed with ZnO 20 nm. [55]. Also Namasivayam et al. and Thati et al. reported that nanoparticles showed enhanced activity with several antibiotics against all the tested *S. aureus* [56, 57]. Several previous studies investigated the prevalence of *mec A, van A* and *cfr* genes among *S. aureus* isolates [34, 58, 59]. However, information about the expression levels of these genes is very little. Therefore, the current study assessed the expression levels of these genes by investigating RNA of S*. aureus* isolates before and after application of ZnO-NPs by quantitative rt-PCR. *Mec* A gene was expressed in 100% of oxacillin resistant isolates and *van A* gene was expressed in (22/39; 50%) of the VRSA isolates. *Cfr* gene expression was detected in (29/34; 85.30%) of LRSA isolates, this high rate could be explained by horizontal spread of *cfr gen*e among different species [60]. *Cfr-*mediated resistance was also identified in 100% of LRSA isolates in previous studies [12]. The expression levels of *mec A, van A* and *cfr* genes after treating the isolates with sub-MIC of ZnO-NPs were significantly reduced with p values; 0.005, 0.002 and 0.001 respectively. Also Kadiyala et al. who examined the effect of ZnO-NP on different genes of *S. aureus* by microarray reported that 375 were significantly down-regulated after application of ZnO-NP [61]. To best of our knowledge, the current study is the first incidence of investigating the effect of ZnO-NPs on levels of gene expression in *S. aureus* isolates in Egypt and Middle East. The current study has also investigated the expression levels of biofilm encoding genes before and after application of ZnO-NPs. The expression of *fnb A, ica A, ica B* and *ica D* genes were investigated; the most frequently expressed genes were *ica A* and *fnb A* (expressed in 100% of isolates), followed by *ica D* gene which has expressed in 82.6% of isolates. However, *ica B* gene has not expressed in the study isolates. The expression of *fnb A, ica A* and *ica D* genes after treating of isolates with sub MIC of ZnO-NPs were significantly reduced and p values were 0.008, 0.005 and 0.005 respectively. That agrees with Abd El-Hamid et al. who demonstrated that the transcriptional levels of *ica*A was remarkably decreased with mean values of fold changes up to 0.15, and Shakerimoghaddam et al., who reported significant reductions 10.2-fold decrease in the gene expression of *icaA* gene expression among *S. aureus* isolates after application of sub MIC ZnO-NPs [45, 63]. However, Gheidar et al. reported that the expression of *icaA* and *icaD* genes in the presence of ZnO-NPs were not significantly reduced compared to the control samples. But, exposure to nanoparticles reduced the expression of *fnbA* and *fnbB* genes from 0.46 to 0.06 [62].

## Conclusion

In this study, promising activities of ZnO-NPs as an antibacterial agent against MRSA, VRSA and LRSA as well as anti-biofilm activity were reported. The study demonstrated that the ZnO-NPs are able to reduce the expression levels of the *ica A, ica D* and *fnb A* genes (the main genes associated with biofilm formation in *S. aureus*) and also reduce the expression levels of the *mec A, van A* and *cfr* genes (the main genes associated with resistance to methicillin, vancomycin and linezolid in *S. aureus*). Finally, we recommend the use of ZnO-NPs for resistant infections. However, further researches must be done to evaluate the safety of ZnO-NPs use in vitro and in vivo.

## Supplementary Information

**Additional file 1**: **Table S1**. Mean relative quantity (RQ) of gene expression before and after application of ZnO NPs.

## Data Availability

All data generated or analyzed during this study are included in this article and Additional file 1.

## Abbreviations

LRSA: Linezolid resistant *S. aureus*
MIC: Minimum inhibitory concentration
MRSA: Methicillin resistant *S. aureus*
ROC curve: Receiver operating characteristic curve
rt-PCR: Reverse transcriptase-polymerase chain reaction
RQ: Relative quantity
VISA: Vancomycin intermediate *S. aureus*
VRSA: Vancomycin resistant *S. aureus*
ZnO-NPs: Zinc oxide nanoparticles
