# Supplementary material for: Effect of ZnO nanoparticles on methicillin, vancomycin, linezolid resistance and biofilm formation in Staphylococcus aureus isolates
Source: Ann Clin Microbiol Antimicrob. 2021 Aug 21;20:54. doi: 10.1186/s12941-021-00459-2 (PMC8379777; doi:10.1186/s12941-021-00459-2)
Supplement: Supplementary file 1 — Additional file 1: Table S1. Mean relative quantity (RQ) of gene expression before and after application of ZnO NPs. [file 12941_2021_459_MOESM1_ESM.docx]

Table S1: Mean relative quantity (RQ) of gene expression before and after application of ZnO- NPs

| **Gene** | **RQ before ZnO**  **Mean(Range)** | **RQ after ZnO**  **Mean(Range)** | **P value** |
| --- | --- | --- | --- |
| *fnbA* | 370 (8:502) | 7.4 (0.014:49) | 0.008 |
| *ica A* | 820.9 (2 :1000) | 5.9 (0.16:8) | 0.005 |
| *ica D* | 42.06 (0.5:77) | 5.14 (0.001:7.4) | 0.005 |
| *mec A* | 23.38 (0.4:66) | 2.59 (0.05:8) | 0.005 |
| *van A* | 4.21 (0.06:6) | 0.07(0.0004:0.2) | 0.002 |
| *cfr* | 76.8 (22:97.3) | 4.81 (1.9:7.3) | 0.001 |

- ***Mann Whitney test for non-parametric quantitative data between the two groups***

****: Significant level at P value < 0.05***
